# Cancer management in the twenty-first century in India

**DOI:** 10.4103/0971-5851.71654

**Published:** 2010

**Authors:** Bhawna Sirohi

**Affiliations:** *Department of Medical and Hemato-Oncology, EBMT Committee on Nuclear Accidents, Artemis Cancer Center, Artemis Health Sciences, Sector 51, Gurgaon, NCR, India E-mail: Bhawnas@artemishealthsciences.com, bhawna.sirohi@btinternet.com*

The key to cancer care in the twenty-first century anywhere in the world, including India, is knowledge, expertise, training, cost-affectivity, targeted therapy, and a balanced lifestyle, for prevention.

Dr. Francesca Morosani Thompson, a New York Orthopedic surgeon was diagnosed with multiple myeloma in the 1980s, and was one of the first few patients to undergo an autologous bone marrow transplant when it was not the standard of care. She went onto write, ‘Going for the cure,’ thus defining twenty-first century medicine for treating cancer patients, which should aim to cure, that is, patients should be able to live with their disease in ‘symbiosis,’ but die of something else.[1]

The cancer strategy for India has to aim at the following components in tandem, to be able to deliver the management for the increasing disease burden.[2]

- Prevention and early detection
- Balanced lifestyle
- Diagnostic services
- Speed of referral to a regional cancer center or specialist unit
- Oncosurgery – radiotherapy – chemotherapy
- Provision for palliative care
- Providing for spiritual needs
- Research
- Cost-effective care

The aim of the doctors in the twenty-first century is not only to improve the survival rates of cancer, but also to be able to effectively prevent cancers related to smoking, infection, diet, and lack of activity, which are preventable, by a robust awareness and education drive, both in the urban and rural areas of India. Doctors should not, ‘practice by the numbers,’ but attend to ‘the science of humanity’. What works for the West – mammograms, regular pap smears, prostate-specific antigen (PSA), and stool occult blood tests – is unlikely to work in India, in view of the limited resources.

All hospitals should offer smoking cessation advice to all patients who walk in through the door. It is also important that every comprehensive cancer center offers cancer patients and also the community as a whole, a service of smoking / tobacco cessation and also promotes tobacco control in schools, working places, and public places.

Simple awareness about maintaining sexual hygiene will go a long way in preventing cervical cancer, which accounts for the majority of the female disease burden in rural India. Screening camps either run by the government or the NGOs, which focus on the simple clinical examination of the oral cavity, breast, and cervix will be the key to diagnosing early cancers and impacting on mortality.

The key to providing balanced care to patients in today’s world, to improve outcomes, is treating them within a multidisciplinary team (MDTs) setting. MDTs lead to improved communication between the professionals involved and patients are, therefore, more likely to receive better continuity and coordination of care through all stages of their disease, as also better advice on the appropriate treatment. MDT working is essential if we are to continue improving the overall experience of cancer patients. This should comprise of not just the doctors involved in patient care, but psychologists, dietitians, physiotherapists and so on, along with the patient support groups.

Cancer is curable if detected early and the aim in advanced stages is also to turn it into a chronic illness by various strategies including targeted therapy. Targeted cancer therapies have changed cancer management, as the treatment is not too toxic and some treatments use the patient’s own immune system to fight the disease. It is an exciting time for those currently working in cancer care. With skilled sequential use of various treatments, it is possible to attain an ever increasing proportion of cancer patients with normal quality of life (QoL), living longer than 10 to 20 years, and for some patients this translates into ‘operational cure’ – patients die of other causes but not cancer.

The ability of the pharmaceutical companies and regulators to fast track the development and approval of newer drugs, and the trend of worldwide collaboration, coupled with patient support agencies, means that more clinical trials delivering new tangible treatments are available for cancer patients. Substantial advances have been made in our understanding of the biology of cancer leading to the availability, in the last decade, of new approved targeted therapies, which are now in common usage, namely Imatinib for leukemia, trastuzumab / lapatinib for breast cancer, and so forth. Targeted therapies, by virtue of being targeted, are less toxic and patients are able to maintain a normal QoL, while being on treatment. QoL can be achieved by treating the disease as intensively as possible, thereby inducing long-term remission (or an ‘operational cure’), or transferring the disease into an indolent course (chronic condition). Time without symptoms, treatment or toxicity (TWIST) favors the use of targeted therapy for managing cancer patients. The progress of treatment for the important steps in the patient’s treatment pathway cannot be considered without at least some attempt at a surrogate assessment of the impact of such treatments upon the QoL of the individual patient.

Gene expression signatures help us guide treatment for various cancers. The genetic makeup of the patient and the tumor influence treatment choices help us in individualizing the treatment for patients. Data on Indian patients need to be generated separately so that it helps us in treating them effectively and in a better manner. For example, a genebased blood test for the detection of breast cancer is being considered to be launched here soon, and the advantage of this is that for a country like India where mammography is not practical to do as a screening test (not cost-effective and also not useful in younger patients), a simple blood test will aid us in the early detection of cancer. Patients will quite likely prefer this and a single laboratory is cheaper than several mammographic facilities.

Access to good quality information is fundamental to making informed choices about personal health and healthcare. Awareness about cancer prevention and treatment among the community, physicians, and rural and urban areas should be the focus of dealing with cancer in the twenty-first century.

Three areas for further progress need prioritized attention, namely:

- Tailoring of sequential targeted treatment for an individual patient
- Optimization of limited financial resources to best deliver this treatment
- How we maximize the QoL for an individual patient
